# Multicompartmental Mesoporous Silica/Polymer Nanostructured Hybrids: Design Capabilities by Integrating Linear and Star-Shaped Block Copolymers

**DOI:** 10.3390/polym12010051

**Published:** 2019-12-31

**Authors:** Zacharoula Iatridi, Kyriaki Evangelatou, Nikolaos Theodorakis, Athina Angelopoulou, Konstantinos Avgoustakis, Constantinos Tsitsilianis

**Affiliations:** 1 Department of Chemical Engineering, University of Patras, 26504 Patras, Greece; ziatridi@gmail.com (Z.I.); kiriaki.evang@gmail.com (K.E.); nikolastheo96@hotmail.com (N.T.); 2 Department of Pharmacy, Medical School, University of Patras, 26504 Patras, Greece; angelopoulouathina@gmail.com (A.A.); avgoust@bioacademy.gr (K.A.)

**Keywords:** organic/inorganic hybrid, mesoporous silica, block copolymer, star-shaped copolymer, monomolecular micelle, LbL technique, multicompartmental, nanostructured carrier

## Abstract

Poly(2-vinyl pyridine)-*b*-poly(ethylene oxide) (P2VP-*b*-PEO) linear diblock copolymer and polystyrene–poly(ethylene oxide) (PS_10_PEO_10_) heteroarm star copolymer were used as building elements to prepare organic–inorganic hybrids. By using the layer-by-layer (LbL) methodology, these elements were integrated on mesoporous silica through non-covalent interactions, namely, ionic and H-bonding. For the latter, tannic acid (TA) was used as an intermediate layer. The deposition of the various layers was monitored by thermogravimetric analysis (TGA), electrophoretic measurements, and confocal microscopy. The final silica hybrid, bearing alternating P2VP-*b*-PEO and PS_10_PEO_10_ star layers was capable of carrying one hydrophilic and two hydrophobic chemical species in distinct compartments. These multicompartmental organic–inorganic hybrids could be used as nanostructured carriers for pH-responsive multiple drug delivery and potential theranostic applications.

## 1. Introduction

The use of mesoporous silica particles (MSPs) as nanocarriers and promising platforms for delivery, diagnostic, or prognostic biomedical applications received tremendous attention from the scientific community. Their remarkable potential application in nanomedicine is due to their unique properties such as well-defined pores with tunable pore size that can encapsulate therapeutic drugs, genes, proteins, and targeting or imaging agents [1,2], large specific surface area, a reactive surface which can be easily functionalized, and biocompatibility [3,4,5,6,7,8]. Nevertheless, MSPs usually suffer from premature release of the encapsulated payload and leakage before reaching the targeted site. To overcome such barriers and confer together other advantageous properties including improved surface functionalities, switchability, responsiveness, or better stability, mesoporous silica nanoparticles (MSNs) are modified by polymers [9,10]. These polymers, which act as gatekeepers to retain the guest molecules inside the silica particles pores, usually respond to external stimuli. The gates open when a stimulus such as pH, temperature, light, ionic strength, magnetic field, or enzyme is applied, allowing the programmable desired payload release. Several studies were devoted to organic/inorganic polymer/MSN-based stimuli-responsive drug delivery systems [11,12], mainly pH-responsive [13,14,15,16], thermo-responsive [17], or responsive to more than one stimulus (e.g., pH- and thermo-responsive [18,19] or photo- and thermo-responsive [20]). In these systems, the polymers are usually covalently bonded onto the MSN surface through grafting methods or polymerization procedures in the presence of silica particles.

The layer-by-layer (LbL) technique is an alternative route to functionalize MSPs with polymers through non-permanent, physical associations, using weak non-covalent bonds, e.g., ionic, hydrogen, and hydrophobic bonds. LbL was proven to be a very promising method for endowing the MSNPs with the desired stimuli-responsive capabilities for controlled release applications. Polyelectrolyte multilayers (PEMs), formed by the ionic interactions of oppositely charged polyelectrolytes, can be adsorbed onto the MSN surface through the LbL technique, turning them into stimulus-responsive systems (pH-responsive), due to their change in structure under different pH conditions. Several studies focused on the assembly of PEM multilayers onto the surface of MNPs for the design of pH-sensitive drug release applications. Some examples of polyelectrolyte pairs used include poly (allylamine hydrochloride) (PAH)/sodium poly(styrene sulfonate) (PSS) [21,22,23] and PAH/poly(acrylic acid) (PAA) [24], or polyelectrolyte pairs based on natural polysaccharides such as chitosan (CHI)/alginate (ALG) [25,26], CHI/dialdehyde starch (DAS) [27], CHI/acacia (ACA) [28], and CHI/hyaluronic acid (HA) [29,30]. All these PEM-MSN systems exhibit controlled storage and release of therapeutic cargo (e.g., drugs, model drugs, imaging agents, biomolecules). The encapsulated cargo release is restrained in neutral or basic condition as the polyelectrolytes cover the MSN surface, while, in acidic conditions, the cargo is released quite easily due to the swelling or coiling of the polyelectrolytes, thus uncovering the MSN pores.

In this communication, we present a paradigm showing the various capabilities of designing MSPs, functionalized by block copolymers, using various interactions through the LbL technique. Two copolymers with different components and architecture were chosen for the coating of mesoporous silica particles. These polymers are a poly(2-vinyl pyridine)-*b*-poly(ethylene oxide) (P2VP-*b*-PEO) linear diblock copolymer and a polystyrene–poly(ethylene oxide) (PS_10_PEO_10_) heteroarm star copolymer [31]. Both bear biocompatible PEO blocks in order to endow the nanocarrier with non-toxicity and biocompatibility. The P2VP-*b*-PEO copolymer was selected to serve as a pore keeper, due to the pH-responsive P2VP block, attributed to the protonation/deprotonation of the pyridine groups (weak cationic polyelectrolyte behavior) [32]. The second polymeric component, the PS_10_PEO_10_ copolymer, bears 10 PS and 10 PEO arms. Thanks to the star-shaped architecture and the asymmetric length of the arms (PS short, PEO long), it tends to form monomolecular spherical micelles in water with a hydrophobic PS core and a hydrophilic PEO corona that preserves the polymer solubility in the aqueous solution [33]. Hence, the hydrophobic PS pockets can be used for the encapsulation of hydrophobic molecules such as drugs and targeting or imaging agents [34].

The LbL technique is an excellent process to integrate both types of the selected polymers onto the MSPs. In the present case, it was based on two different mechanisms. The first layer of P2VP-*b*-PEO, coating the surface of the SiO_2_ particles, interacts with the silica surface through ionic interactions between the negatively charged silica and the positively charged P2VP block. The next layers are deposited through the formation of hydrogen bonds between the PEO blocks of the P2VP-*b*-PEO and PS_10_PEO_10_ copolymers using intermediate tannic acid (TA) layers. TA is a polyphenol with characteristic antioxidant, anticarcinogenic, antimicrobial, and antibacterial properties that can interact with various molecules through several mechanisms (electrostatic interactions, hydrophobic interactions, hydrogen bonding, etc.) [35,36,37,38,39]. It was used with the LbL technique forming multilayer assemblies via hydrogen bonding with pH-responsive, thermo-responsive, or neutral polymers including TA/poly(allylamine) (PAH) [36], TA/poly(*N*-vinylpyrrolidone) (PVPON) [40,41,42,43], TA/poly(ethylene oxide) (PEO), TA/poly(ethylene glycol) (PEG) [44,45,46], TA/poly(*N*-vinylcaprolactam) (PVCL) [37], and TA/poly(*N*-isopropylacrylamide) (PNIPAM) [37].

Therefore, by integrating pH-responsive block copolymers and monomolecular star-based micelles into the MSP, novel functional materials can emerge as multicompartmental carriers for multiple drug delivery and theranostic potential applications.

## 2. Experimental Section

### 2.1. Materials

The linear diblock P2VP-*b*-PEO and the heteroarm star PS_10_-PEO_10_ copolymers were synthesized in the lab (see Supplementary Materials). The detailed molecular characteristics of the copolymers are summarized in Table S1 (Supplementary Materials). Mesoporous silica particles with a diameter of 3.0 µm were obtained from Sigma-Aldrich (Athens, Greece). The characteristics of the particles, as given from the provider, are presented in Table S2 (Supplementary Materials). Tannic acid (TA, molecular weight (Mw) = 1700 Da), calcein, Nile Red, and pyrene were products of Sigma-Aldrich (Athens, Greece). Standard solutions of hydrochloric acid (HCl) and sodium hydroxide (NaOH) were used as purchased from Panreac (Athens, Greece) without further purification. The organic solvent tetrahydrofuran (THF) was obtained from Sigma-Aldrich (Athens, Greece). Ultrapure water was obtained by means of an ELGA Medica-R7/15 device (ELGA Labwater, Athens, Greece).

### 2.2. Preparation of SiO_2_@P2VP-b-PEO Particles

Three sets of SiO_2_@P2VP-*b*-PEO particles were fabricated following a simple method. Briefly, P2VP-*b*-PEO was dissolved in HCl 0.001 M (pH 3.0) at a concentration of 8 mg/mL. Then, a powder of bare SiO_2_ (30 mg) was added in an adequate volume (from ~3 to 5 mL) of the P2VP-*b*-PEO solution, and the dispersion was stirred vigorously at room temperature for 15 min, followed by two centrifugation (3000 rpm for 2 min)/wash (with HCl 0.001 M) cycles. The theoretical m_polymer_/m_SiO2_ ratios that were used for the preparation of the three SiO_2_@P2VP-*b*-PEO particles are presented in Table 1. The SiO_2_@P2VP-*b*-PEO particles were separated as a precipitate and dried in the oven. The absorption percentage of the P2VP-*b*-PEO onto the SiO_2_ particles was analyzed by thermogravimetric analysis (TGA) and ultraviolet–visible light (UV–Vis) spectroscopy.

### 2.3. Encapsulation of Nile Red or Pyrene in PS_10_-PEO_10_ Micelles

PS_10_-PEO_10_ was firstly dissolved in a small amount of THF, followed by the addition of ultrapure water under stirring. THF was further removed from the polymer solution by heating in the oven. The polymer concentration was fixed at 0.2 mg/mL. At a next step, Nile Red was dissolved in THF with a concentration of 0.5 mg/mL. Then, 60 µL of Nile Red solution was added to 10 mL of PS_10_-PEO_10_ solution dropwise with constant stirring for 1 h. Afterward, the PS_10_-PEO_10_/Nile Red solution was dialyzed against ultrapure water for two days with repeated changes of water in order to remove excess Nile Red molecules in the solution. The final PS_10_-PEO_10_/Nile Red solution was kept in the dark. The same procedure was followed for the preparation of pyrene-encapsulated PS_10_-PEO_10_ micelles.

### 2.4. Preparation of SiO_2_@P2VP-b-PEO@TA@PS_10_-PEO_10_ Particles

Aqueous solution of PS_10_-PEO_10_ with or without encapsulated Nile Red or pyrene had a concentration of 0.2 mg/mL. TA was dissolved in 0.1 M NaCl solution at the concentration of 0.2 mg/mL. The pH of the TA solution was adjusted to pH 5.0 with NaOH. SiO_2_@P2VP-*b*-PEO particles, prepared as described above, were incubated in 1.5 mL of TA solution for 15 min, followed by two centrifugation (3000 rpm for 2 min)/wash (aqueous solution with fixed pH 5.0) cycles. Afterward, 1.5 mL of PS_10_-PEO_10_ aqueous solution was added, and 15 min was allowed for adsorption, followed by two centrifugation/wash cycles with water. The adsorption steps were repeated until the desired number of layers was built on silica particles.

### 2.5. Loading and Release of Calcein in SiO_2_@P2VP-b-PEO Particles

Firstly, calcein was dispersed in ultrapure water (3 mg/mL) with a fixed pH 3.0 using HCl standard solution. The solution was magnetically stirred in the dark and at room temperature for 24 h. Afterward, 50 mg of SiO_2_@P2VP-*b*-PEO powder was dispersed in 11 mL of the calcein solution and stirred overnight in the dark. At a next step, three centrifugation (3000 rpm for 2 min)/wash (with HCl 0.001 M) cycles were performed, and the final SiO_2_@P2VP-*b*-PEO@calcein particles were separated as a precipitate and dried.

For the determination of the encapsulated amount of calcein in SiO_2_@P2VP-*b*-PEO particles, a calibration curve of aqueous calcein solutions (various concentrations) was prepared through UV–Vis absorbance measurements of standard calcein solutions (Figure S3, Supplementary Materials). The encapsulation was calculated by measuring the absorbance of the supernatant solutions obtained from the centrifugation/wash cycles at 485 nm. The theoretical loading, loading capacity, or loading content and the entrapment efficiency of calcein in the SiO_2_@P2VP-*b*-PEO particles were calculated using the following equations:

(1) $$Lth\%\;=\;\frac{W_{0}}{W_{C}+W_{0}}\;\times \;100,$$

(2) $$LC\%\;=\;\frac{W}{W_{C}+W}\;\times \;100,$$

(3) $$ΕΕ\%\;=\;\frac{W}{W_{0}}\;\times \;100,$$

where W, W_C_, and W_0_ are the amount of entrapped calcein according to UV–Vis spectroscopy, the amount of SiO_2_@P2VP-*b*-PEO@calcein particles, and the amount of initially added calcein, respectively. The theoretical loading was 39.8%, and it was found that the loading capacity of the SiO_2_@P2VP-*b*-PEO@calcein particles was 16.7%, while the entrapment efficiency was 30.3%.

For the pH-responsive calcein release test, phosphate buffer (PB) solutions (10 mM) with pH 5.0, 6.0, and 7.4 were firstly prepared. Then, ~2 mg of SiO_2_@P2VP-*b*-PEO@calcein was dispersed in 1 mL of deionized water, and the dispersion was transferred into a dialysis bag (molecular weight cutoff 14 kDa). Subsequently, the dialysis bag was placed in 13 mL of buffer solution with pH 5.0, 6.0, or 7.4 at 37 °C and shaken in the dark at 100 rpm. At timed intervals, the entire volume of the outer buffer solution was removed and renewed with 13 mL of fresh medium. The released calcein was calculated by UV–Vis spectroscopy using the absorbance intensity at 485 nm and a calibration curve of calcein at PB buffers of pH 5.0, 6.0, and 7.4 (Figures S4–S6, Supplementary Materials).

### 2.6. Techniques

Quantitative analysis in order to confirm the exact mass of each layer was done via thermogravimetric analysis (TGA) using a Discovery TGA™ equipment (TA Instruments, New Castle, DE, USA) under nitrogen atmosphere with a flow rate of 40 mL/min. Firstly, 5–7 mg of each sample was deposited in an alumina ceramic crucible. Then, an isothermal process was carried out at 40 °C. Temperature varied from 40 to 800 °C, and the heating rate used was 20 °C/min. UV–Vis spectra were recorded using a double beam HITACHI U-2001 UV–Vis spectrophotometer (HITACHI, Athens, Greece). Aqueous solutions of the samples were put into quartz cuvettes with a 1-cm optical path. The surface potentials of bare and coated silica particles were measured at 25 °C on a zeta potential analyzer (Malvern Nano Zetasizer, Malvern, Athens, Greece) equipped with an He–Ne laser at 633 nm. For these measurements, approximately 1-mL aqueous dispersions were used, and the zeta potential was calculated by the Smoluchowski relation. Each zeta potential value was obtained by averaging three independent measurements of 40 sub-runs each. The morphology of the bare and the LbL-coated SiO_2_ particles was observed by TEM using a JEM-2100 microscope (JEOL, Haarlemmermeer, The Netherlands) operating at 200 kV. The specimens were prepared by dispersing the powdered samples in ultrapure water (0.1 mg/mL) and depositing some drops of the dispersion on a carbon grid. The grids were left to stand at room temperature until full evaporation of the diluent. The confocal images of the LbL-coated silica particles were obtained with a Leica SP5 Confocal Laser Scanning Microscope (CLSM) (Leica Microsystems, Athens, Greece) equipped with an argon laser source and a CApochromat 63× oil immersion objective. A drop of the polymer-coated SiO_2_ particle suspension was deposited on a microscope slide and left to dry. Then, the slide was mounted with Mowiol 4-88 (Sigma Aldrich, Athens, Greece).

## 3. Results and Discussion

The mesoporous silica microparticles were covered by successive polymeric layers using the LbL method in order to prepare potential pH-responsive nanostructured drug carriers. For this reason, two different copolymers were used, a linear pH-responsive diblock copolymer and a star-shaped amphiphilic copolymer. The molecular characteristics of the copolymers are presented in Table S1 (Supplementary Materials).

### 3.1. SiO_2_@P2VP-b-PEO Hybrids

Initially, SiO_2_@P2VP-*b*-PEO hybrids were designed using a P2VP-*b*-PEO diblock copolymer as the gatekeeper of the mesoporous silica. The hybrid was prepared by physisorption of the P2VP-*b*-PEO copolymer at the SiO_2_ particle surface. From electrophoresis measurements of dispersions of the SiO_2_ particles in aqueous solutions with fixed pH values varying from 2.0 to 7.5 (using HCl 1 M or NaOH 1 M) (Figure S1, Supplementary Materials), it was found that the mesoporous silica particles were negatively charged above pH 2.0 with decreasing zeta potential, in accordance with the literature [47]. On the other hand, the pH-responsive diblock copolymer P2VP-*b*-PEO bears positive charges in an acidic environment due to the protonation of the pyridine group of P2VP which exhibits a pKa at pH 5 (50% degree of ionization). Thus, the first layer coating of the SiO_2_ particles with P2VP-*b*-PEO was attempted in an aqueous solution with pH 3.0 where both constituents bore charged moieties (Figure S1, Supplementary Materials). Thus, the adsorption of the copolymer onto the silica surface was due to the ionic interactions between the negatively charged particle surface and the positively charged moieties of the copolymer P2VP block. Three samples of SiO_2_@P2VP-*b*-PEO were prepared, differing in the theoretical m_polymer_/m_SiO2_ ratio (Table 1).

**Figure 1:**
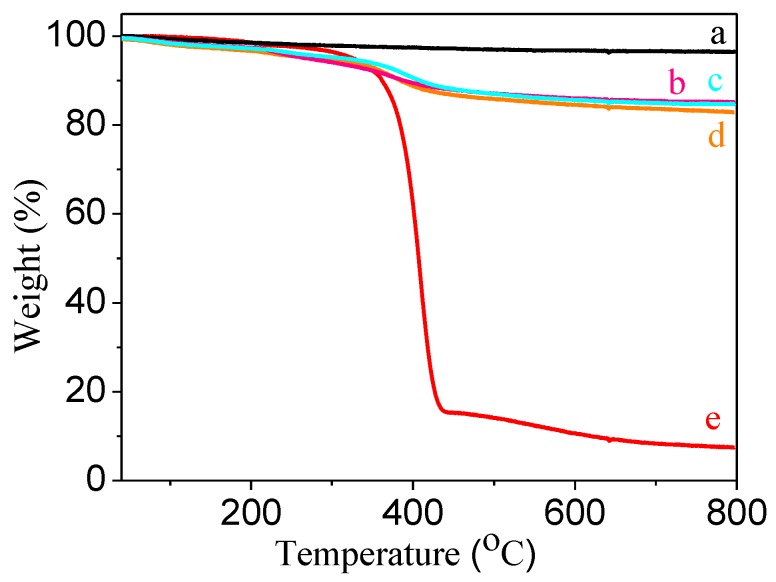
Thermogravimetric analysis (TGA) curves of (**a**) the bare SiO_2_ (black line), (**b**) SiO_2_@poly(2-vinyl pyridine)-*b*-poly(ethylene oxide) (P2VP-*b*-PEO)_1 (pink line), (**c**) SiO_2_@P2VP-*b*-PEO_2 (cyan line), (**d**) SiO_2_@P2VP-*b*-PEO_3 (orange line), and (**e**) P2VP-*b*-PEO (red line).

The TGA curves of the bare SiO_2_ particles, samples of silica particles coated with P2VP-*b*-PEO, and the polymer P2VP-*b*-PEO are presented in Figure 1. For the bare silica particles (Figure 1a), it can be observed that the TGA curve was almost horizontal, suggesting the lack of organic residues in the SiO_2_ particles. On the contrary, the pure polymer P2VP-*b*-PEO sample (Figure 1e) exhibited a remarkably high mass loss (≥90%) for temperatures higher than 400 °C, representative of the behavior of polymers and other organic substances. All three SiO_2_@P2VP-*b*-PEO samples showed an intermediate behavior (Figure 1b–d). Their TGA curves declined upon increasing temperature, owing to the mass loss of the organic layer, proving that the surface of the silica particles was indeed successfully coated with polymer. In fact, the SiO_2_@P2VP-*b*-PEO samples had a mass loss of ~15%. From this technique, the quantitative analysis of the adsorbed P2VP-*b*-PEO copolymer onto the silica surface was performed. At the same time, the amount of copolymer adsorbed onto the silica was determined with UV–Vis spectroscopy through a calibration curve of the P2VP-*b*-PEO copolymer at aqueous solutions of pH 3.0 (Figure S2, Supplementary Materials). More specifically, the concentration of the non-adsorbed copolymer in the supernatant solutions after the centrifugation/wash cycles was calculated through the calibration curve. Finally, the adsorbed copolymer was found, knowing the initial amount of copolymer added and the non-adsorbed amount. The results of the quantitative analysis from both TGA and UV–Vis spectroscopy are tabulated in Table 1. As can be seen, these two different techniques had a low deviation (~5%), implying the good agreement between them.

**Figure 2:**
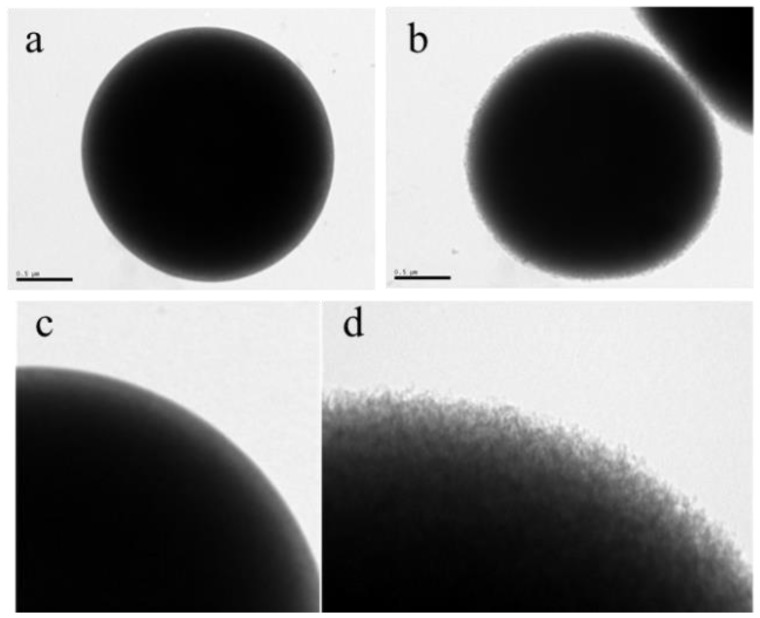
TEM images of (**a**,**c**) bare SiO_2_, and (**b**,**d**) SiO_2_@P2VP-*b*-PEO particle hybrid. Scale bars in (**a**,**b**) 500 nm. The (**c**,**d**) images are magnifications of the corresponding (**a**,**b**) images, respectively.

The morphology of the P2VP-*b*-PEO-coated silica particles was investigated by TEM (Figure 2). As can be seen in Figure 2a, the bare SiO_2_ particles were spherical particles with a very smooth edge. In Figure 2b, a TEM image of the SiO_2_@P2VP-*b*-PEO particle hybrid is presented. As can be more clearly seen in the magnified area of Figure 2b,c, the surface of these particles clearly differs from that of the smooth SiO_2_ particles. An increased roughness of the surface appeared, verifying the successful deposition of the P2VP-*b*-PEO polymer coating onto the SiO_2_. Due to the diblock architecture and the involved interactions between the SiO_2_ and the P2VP blocks, the PEO block chains covered the outer layer of the SiO_2_@P2VP-*b*-PEO hybrids. Yet, the adsorbed polymeric layer appeared uniform without defects. Due to the diblock architecture, the incompatibility of the blocks, and the involved interactions between the SiO_2_ and the P2VP blocks, the PEO chains covered the outer layer of the SiO_2_@P2VP-*b*-PEO hybrids. The stability of the adsorbed layer was checked by redispersing the particles in aqueous media of different pH. After rigorous stirring and centrifugation, possible delamination effects were evaluated by determining the amount of the polymer left in the supernatant. The polymer loss was lower than 3.6 wt.% for all pH values investigated (Figure S7, Supplementary Materials).

**Figure 3:**
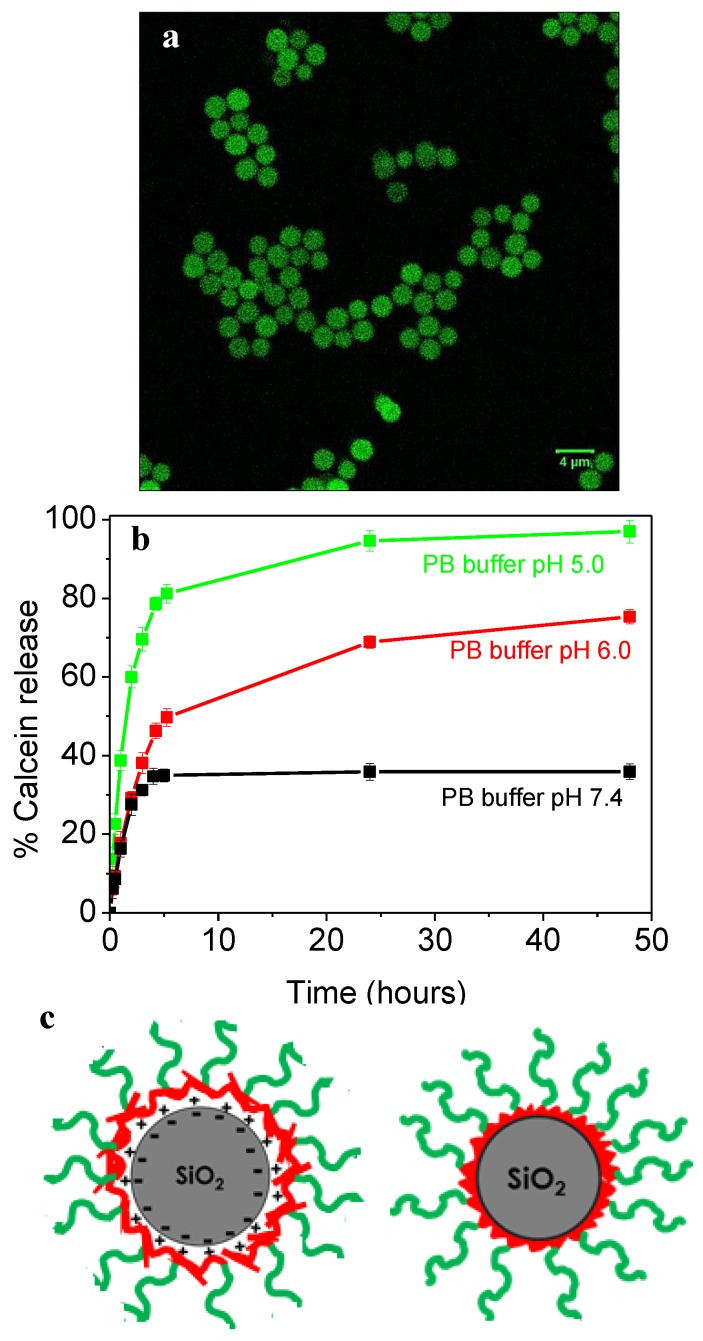
(**a**) Confocal microscopy image of calcein-loaded SiO_2_@P2VP-*b*-PEO@calcein particles; (**b**) pH-triggered release profile of calcein from SiO_2_@P2VP-*b*-PEO@calcein particles; (**c**) schematic representation of an SiO_2_@P2VP-*b*-PEO microparticle in acidic (left) and basic (right) pH environments. The red color represents P2VP and the green color represents PEO. Scale bar in (**a**): 4 μm.

In order to evaluate the function of the P2VP blocks as a pH-responsive gatekeeper, calcein (fluorescent molecule) was encapsulated within the pores of the SiO_2_ particles followed by controlled release experiments at various pH values. The calcein encapsulation was qualitatively confirmed by means of confocal microscopy. In Figure 3a, the confocal image of calcein-loaded SiO_2_@P2VP-*b*-PEO particles is presented. As it can be seen, the calcein was successfully encapsulated (green color) in the silica particles.

Calcein release from the SiO_2_@P2VP-*b*-PEO@calcein particles was investigated in three different buffers with pH 5.0, 6.0, and 7.4 (corresponding roughly to the pH of lysosomes, the pH of cancer cells, and physiological pH, respectively). The samples were shaken in a shaker at a constant temperature of 37 °C. The results of calcein release at pH 5.0, 6.0, and 7.4 are presented in Figure 3b. From the diagram, it can be clearly seen that the system exhibited pH sensitivity with pH-controlled release properties. At physiological pH (pH 7.4), the release rate was relatively low, and the released amount reached 36% in the first 5 h, while no further release observed for more than 40 h. The released amount might have arisen from a portion of calcein adsorbed on the SiO_2_/P2VP surface layer. This finding indicates a satisfactory storage ability and the gatekeeper effect of the P2VP-*b*-PEO layer. At this pH value, the 2VP groups of the P2VP-*b*-PEO diblock copolymer are completely deprotonated (zero zeta potential) and hydrophobic, thus adopting a globule chain conformation (Figure 3c right), sealing to a large extent the pores of the SiO_2_ particles and keeping a high amount of calcein retained within the particles. When the pH value was lowered to 6.0, the release rate increased and 75% of calcein was released in 48 h. At this pH, the P2VP-*b*-PEO chains adopt a less compact conformation due to the partial (low) ionization of P2VP, thus blocking silica particle pores to a lesser extent and allowing leakage of higher amounts of calcein from the particles. At an even lower pH value of 5.0, the amount of released calcein was 96% in 48 h. This high release rate is attributed to further protonation of the 2VP groups (50% ionization) in the P2VP-*b*-PEO copolymer. As the ionization of the P2VP blocks increases, repulsive interactions along the chains impose expanded conformations (Figure 3c left). Hence, the pores of the silica particles become uncapped and the encapsulated model drug can easily diffuse into the release medium from the mesoporous canals.

### 3.2. SiO_2_@P2VP-b-PEO@TA@PS_10_-PEO_10_ Nanostructured Hybrids

**Figure 4:**
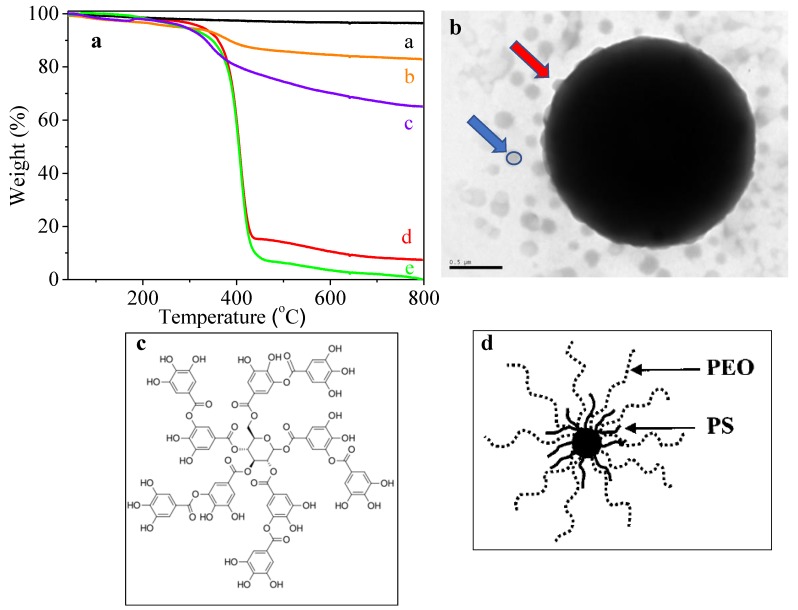
(**a**) TGA curves of (a) the bare SiO_2_ (black line), (b) SiO_2_@P2VP-*b*-PEO (orange line), (c) SiO_2_@P2VP-*b*-PEO@tannic acid (TA)@ polystyrene–poly(ethylene oxide) (PS_10_-PEO_10_) (purple line), (d) P2VP-*b*-PEO (red line), and (e) PS_10_-PEO_10_ (green line); (**b**) TEM image of an SiO_2_@P2VP-*b*-PEO@TA@PS_10_-PEO_10_ particle, scale bar: 500 nm; the blue and red arrows indicate the free and adsorbed PS_10_-PEO_10_ micelles, respectively. (**c**) Molecule of tannic acid; (**d**) a monomolecular micelle of the star-shaped PS_10_-PEO_10_ copolymer.

**Scheme 1:**
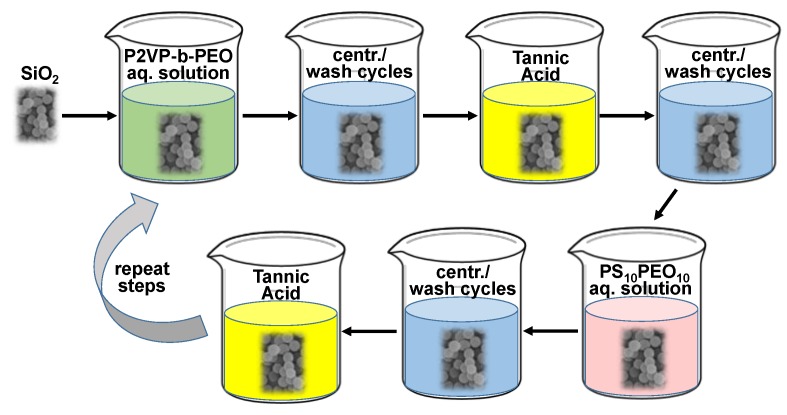
Schematic representation of the layer-by-layer (LbL) method for the fabrication of SiO_2_/copolymer hybrids.

As a next step, the SiO_2_@P2VP-*b*-PEO particles were enriched with another outer layer, constituting a PS_10_-PEO_10_ heteroarm star copolymer. PS_10_-PEO_10_ was chosen as the second copolymer because of its star-shaped architecture and its amphiphilic character. It mainly forms monomolecular micelles (Figure 4d) in water with a hydrophilic PEO corona and a hydrophobic PS core, which can be used as a hydrophobic pocket for encapsulating small hydrophobic molecules (e.g., drugs). The LbL technique was used to fabricate silica particles coated with alternating layers of the diblock P2VP-*b*-PEO copolymer and the PS_10_-PEO_10_ star copolymer. The P2VP-*b*-PEO layer was adsorbed firstly onto the silica surface through ionic interactions as described previously. Tannic acid, the chemical structure of which is presented in Figure 4c, was used as an intermediate layer connecting the two polymeric layers P2VP-*b*-PEO and PS_10_-PEO_10_ through hydrogen bonding between the PEO blocks and arms. Scheme 1 represents the LbL procedure used for the fabrication of the SiO_2_/copolymer hybrids.

In Figure 4, the TGA curves of the bare SiO_2_ particles and the LbL-coated silica particles SiO_2_@P2VP-*b*-PEO and SiO_2_@P2VP-*b*-PEO@TA@PS_10_-PEO_10_ are presented. In the same figure, the TGA curves of the polymers (P2VP-*b*-PEO and PS_10_-PEO_10_) that were used for the LbL coating of the silica particles are shown (Figure 4a(d,e)). As already seen in Figure 1, the bare silica particles (Figure 4a) had an almost horizontal TGA curve, which implies the lack of organic residues in the SiO_2_ particles. The pure polymers displayed high mass loss (≥90%) at T > 400 °C, representative of the behavior of polymers and other organic substances. The SiO_2_@P2VP-*b*-PEO and SiO_2_@P2VP-*b*-PEO@TA@PS_10_-PEO_10_ samples showed an intermediate behavior. Their TGA curves declined upon increasing temperature, owing to the mass loss of the organic layers, indicating that the surface of the silica particles was indeed successfully coated with polymers. The SiO_2_@P2VP-*b*-PEO particles (Figure 4b) had a mass loss of ~15%, while the SiO_2_@P2VP-*b*-PEO@TA@PS_10_-PEO_10_ particles (Figure 4b) exhibited a higher mass loss of ~35%. Hence, the 20% higher mass loss was attributed to the TA/PS_10_-PEO_10_ layers.

From the TEM image of an SiO_2_@P2VP-*b*-PEO@TA@PS_10_-PEO_10_ particle in Figure 4b, it can be clearly seen that spherical monomolecular micelles of the PS_10_-PEO_10_ copolymer, of about 116 nm in diameter, were adsorbed onto the surface of the silica particles. Several non-adsorbed micelles are also visible, in the vicinity of the particle. This image verifies the successful fabrication of the SiO_2_/copolymer hybrids.

In an attempt to develop a nanocarrier with the encapsulation of multiple therapeutic agents and delivery capabilities by integrating a pH-responsive block copolymer and monomolecular star-based micelles to the mesoporous SiO_2_ particles, we proceeded in the fabrication of SiO_2_@calcein@P2VP-*b*-PEO@TA@PS_10_-PEO_10_/Nile Red, where the hydrophilic model drug calcein was encapsulated in the interior of the silica particles, followed by the coating of the silica surface through the LbL method. The first polymeric layer was by the diblock copolymer P2VP-*b*-PEO, an intermediate TA layer was added through hydrogen bonding, and a second polymeric layer of star-shaped monomolecular micelles formed by the PS_10_-PEO_10_ in aqueous media was adsorbed. The hydrophobic associations formed by the hydrophobic PS arms of the amphiphilic star copolymer served as hydrophobic pockets that could encapsulate the hydrophobic dye Nile Red.

**Figure 5:**
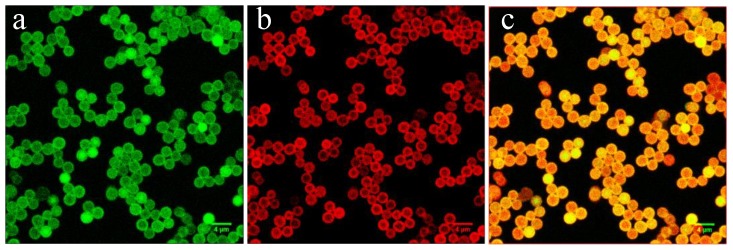
Confocal microscopy images of SiO_2_@calcein@P2VP-*b*-PEO@TA@PS_10_-PEO_10_/Nile Red particles. In image (**a**), the green color from calcein is shown (excitation 495 nm/emission 519 nm). In image (**b**), the red color from Nile Red (excitation 550 nm/emission 573 nm) is shown. Image (**c**) is the merged image of green (**a**) and red (**b**) calcein and Nile Red, respectively. Scale bars: 4 μm.

Indeed, calcein and Nile Red encapsulation in the interior of the particles and the PS_10_-PEO_10_ polymer coating of the mesoporous silica particles, respectively, was confirmed by optical observation by means of confocal microscopy. In Figure 5, the images of SiO_2_@calcein@P2VP-*b*-PEO@TA@PS_10_-PEO_10_/Nile Red particles are shown. The green color in Figure 5a and the red color in Figure 5b are attributed to calcein and Nile Red fluorescence, respectively, and these colors are indicative of the fact that the SiO_2_ particles encapsulated calcein, and that they were effectively coated with the PS_10_-PEO_10_/Nile Red polymer layer. After merging the images of Figure 5a,b, the green and red fluorescences completely overlapped (Figure 5c), which further confirmed the PS_10_-PEO_10_/Nile Red layer on the silica particles.

### 3.3. SiO_2_ Hybrids with Alternating P2VP-b-PEO and PS_10_-PEO_10_ Layers

**Figure 6:**
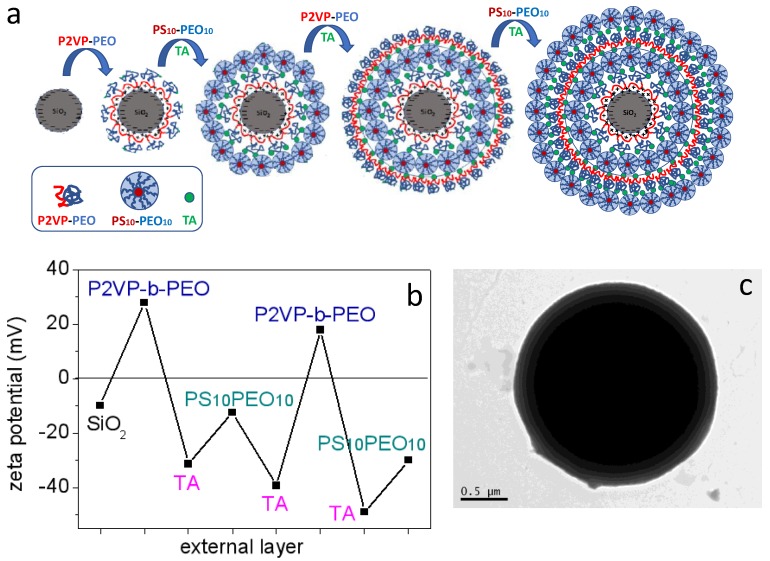
(**a**) Schematic representation of the LbL fabrication of SiO_2_@P2VP-*b*-PEO@TA@PS_10_-PEO_10_@TA@P2VP-*b*-PEO@TA@PS_10_-PEO_10_ hybrid (the various building blocks, P2VP-*b*-PEO, PS_10_-PEO_10_ stars, and TA (tannic acid), are also illustrated to the lower left). (**b**) Evolution of zeta potential during deposition of multilayer on surfaces of silica particles in aqueous solution. (**c**) TEM image of the hybrid microparticle.

In an attempt to demonstrate the design capabilities toward multilayered multicompartmental mesoporous SiO_2_ hybrids, capable of carrying multiple chemical species in different nanostructured compartments, we proceeded to fabricate SiO_2_ particles with alternating P2VP-*b*-PEO and PS_10_-PEO_10_ layers. As an example, SiO_2_@P2VP-*b*-PEO@TA@PS_10_-PEO_10_@TA@P2VP-*b*-PEO@TA@PS_10_-PEO_10_ (see schematic structure in Figure 6a) nanostructured microparticles were prepared following the LbL procedure described in Scheme 1. The successive deposition of the various layers onto SiO_2_ was monitored by electrophoretic measurements. The zeta potential evolution of the silica particles after the adsorption of each layer is presented in Figure 6b. Initially, when dispersed in an aqueous solution with a fixed pH value of pH 3.0, the bare SiO_2_ particles had a negative zeta potential of −10 mV. The zeta potential of the silica particles after the interaction with the P2VP-*b*-PEO polymer was changed from negative to positive, obtaining a value of +28 mV. This is due to the positive charges of the P2VP blocks that overcompensate for the negative charges of the silica particles, which suggests the effective adsorption of a polyelectrolyte layer. Such surface charge reversal is typical in electrostatic LbL assemblies. Next, an aqueous solution of TA was added, resulting in a hybrid system with a negative zeta potential of −31 mV, indicative of the successful deposition of a TA layer. TA interacted with the PEO blocks of the P2VP-*b*-PEO adsorbed copolymer, which should be located in the outer layer of the SiO_2_@P2VP-*b*-PEO particles, through hydrogen bonding. The zeta potential was measured after redispersion of the hybrid at pH 6.5. The relatively high negative value of the TA outer layer could be attributed to the ionized phenolic groups of TA molecules. Similar values were reported in hydrogen-bonded tannic acid LbL assemblies [40].

Afterward, the PS_10_-PEO_10_ solution was added, and the adsorption of the new layer onto the hybrid particle surface was proven as the zeta potential changed from −31 mV to −12 mV. In this case, the PEO arms of PS_10_-PEO_10_ formed hydrogen bonds with TA. Again, after addition of TA to the system, the zeta potential became more negative, −39 mV, confirming the TA deposition through hydrogen bonding. Next, P2VP-*b*-PEO was added, and the zeta potential exhibited a positive value of +18 mV, indicating that ionic interactions again came into play. Evidently, the positively charged P2VP blocks overcompensated for the negative charges of TA. The LbL procedure was continued with another bilayer (TA and PS_10_-PEO_10_). Reversal from +18 mV to −49 mV and finally to −29 mV confirmed the successful deposition of this bilayer. Therefore, by monitoring the zeta potential values of silica hybrids after the adsorption of each layer, the successful deposition of the different copolymers on the particles surface was confirmed (Figure 6a), which was achieved by a combination of ionic interactions and hydrogen bonding with the aid of TA. In Figure 6c, a TEM image of an isolated multilayered hybrid microparticle is also demonstrated, showing almost uniform deposition of the various layers with limited defects.

**Figure 7:**
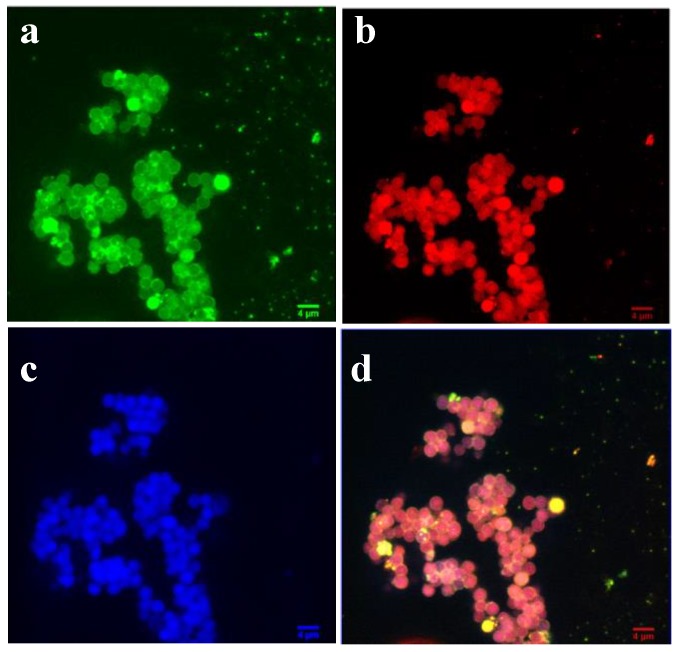
Confocal microscopy images of SiO_2_(calcein)@P2VP-*b*-PEO@TA@PS_10_-PEO_10_(NileRed)@TA@P2VP-*b*-PEO@TA@PS_10_-PEO_10_(pyrene) particles. In images (**a**–**c**), the green channel from calcein (excitation 495 nm/emission 519 nm), the red channel from Nile Red (excitation 550 nm/emission 573 nm), and the blue channel from pyrene (excitation 359 nm/emission 457 nm) are shown, respectively. Image (**d**) is the merged channel from the green, red, and blue channels of calcein, Nile Red, and pyrene, respectively.

In an effort to confirm the successful preparation of the multilayered SiO_2_@P2VP-*b*-PEO@TA@PS_10_-PEO_10_@TA@P2VP-*b*-PEO@TA@PS_10_-PEO_10_ (as seen in Figure 6), and to indicate the compartmentalization of the whole particle hybrid, confocal microscopy was used, by loading the various compartments with three different molecules. Thus, calcein was encapsulated in the interior of the SiO_2_ particles, and two sets of PS_10_-PEO_10_ micelles were loaded with two different hydrophobic dyes, Nile Red and pyrene, prior to the LbL procedure. The final loaded particle structure, resulting from the successive deposition of the layers, was SiO_2_(calcein)@P2VP-*b*-PEO@TA@PS_10_-PEO_10_(Nile Red)@TA@P2VP-*b*-PEO@TA@PS_10_-PEO_10_(pyrene). Figure 7 shows the confocal images of the particles, where in fact the silica core contained calcein while the surface was coated by two successive layers of the PS_10_-PEO_10_ micelles encapsulating Nile Red and pyrene (PS_10_-PEO_10_(Nile Red) and PS_10_-PEO_10_(pyrene)) in their hydrophobic pockets. The green fluorescence (Figure 7a), red fluorescence (Figure 7b), and blue fluorescence (Figure 7c) were accredited to calcein, Nile Red, and pyrene, respectively. The presence of all three colors indicates the effective encapsulation of the dyes in different compartments and distinct layers, i.e., the hydrophilic calcein in the silica cores, as well as the hydrophobic pyrene and Nile Red in the hydrophobic pockets of the PS_10_-PEO_10_ star micelles in different successive layers. The merge image in Figure 7d shows that the three fluorescences completely overlapped, giving a light-purple color as expected from the LbL structure of the hybrid.

We should mention here an interesting future of these multilayered organic–inorganic hybrid microparticles. The polymeric layers constitute nanostructured micellar structures of PS_10_PEO_10_ stars. It is known that these amphiphilic heteroarm star copolymers form spherical, mainly unimolecular, micelles [48] in which the PS arms are segregated in the center of the star-core forming spherical hydrophobic domains (hydrophobic pockets), while the long PEO arms form the surrounding corona (core–shell structure). Due to the high hydrophobicity of the PS chains, the formed micellar structures are kinetically “frozen” and, thus, stable. Provided that the deposition of the heteroarm stars takes place under conditions favoring micellar nanostructuration, the star-micelles should be integrated into the particle surface as segregated nanoparticles, preserving the spherical domains of the PS cores.

## 4. Conclusions

In this work, organic–inorganic hybrids constituting mesoporous silica, layered by linear and star-shaped PEO-based block copolymers, were prepared using the LbL methodology. In the present paradigm, the design capabilities of using various types of nanostructured block copolymers, as building elements, and various non-covalent interactions among them (i.e., ionic and hydrogen bonding) to create multicompartmental multilayered mesoporous silica were demonstrated. The P2VP-*b*-PEO diblock copolymer was used as the pH-responsive gatekeeper, and the PS_10_-PEO_10_ star was used as monomolecular micelles bearing well-protected hydrophobic compartments. By integrating these building elements into the mesoporous SiO_2_ in alternating layers of P2VP-*b*-PEO and PS_10_-PEO_10_, the resulting hybrid particles were capable of carrying three different chemical species, one hydrophilic compound within the mesoporous silica and two different hydrophobic species in different compartments at distinct layers. These types of multicompartmental nanostructured carriers could be suitably designed for biomedical applications such as stimuli (pH)-responsive multiple drug delivery systems and theranostics.

## Figures and Tables

**Table 1:** Quantitative analysis of the adsorbed poly(2-vinyl pyridine)-*b*-poly(ethylene oxide) (P2VP-*b*-PEO) through ultraviolet–visible light (UV–Vis) spectroscopy and thermogravimetric analysis (TGA).

Sample	Theoretical m_polymer_/m_SiO2_ (%)	m_polymer_/m_SiO2_ (%) from UV–Vis	m_polymer_/m_SiO2_ (%) from TGA
SiO_2_@P2VP-*b*-PEO_1	90.66	12.35	11.7
SiO_2_@P2VP-*b*-PEO_2	87.66	12.87	12.2
SiO_2_@P2VP-*b*-PEO_3	137.33	15.2	14.4

## Supplementary Materials

The following are available online at https://www.mdpi.com/2073-4360/12/1/51/s1: Table S1: Molecular characteristics of the polymers; Table S2: Characteristics of the SiO_2_ particles; Figure S1: Zeta potential of aqueous dispersion of SiO_2_ at different pH; Figure S2: (a) UV–Vis spectra of aqueous P2VP-*b*-PEO solutions at a fixed pH 3.0; (b) calibration curve of the P2VP-*b*-PEO at pH 3.0; Figure S3: (a) Emission spectra of aqueous calcein solutions at a fixed pH 3.0; (b) calibration curve of calcein at pH 3.0; Figure S4: Calibration curve of calcein at PB buffer pH 5.0; Figure S5: Calibration curve of calcein at PB buffer pH 6.0; Figure S6: Calibration curve of calcein at PB buffer pH 7.4; Figure S7: Loss (wt.%) of adsorbed copolymer versus washing cycle (redispersion–stirring–centrifugation–separation) at various pH.

## References

[1] Chen N.T., Cheng S.H., Souris J.S., Chen C.T., Mou C.Y., Lo L.W. (2013). Theranostic Applications of Mesoporous Silica Nanoparticles and Their Organic/Inorganic Hybrids. J. Mater. Chem. B.

[2] Tang F., Li L., Chen D. (2012). Mesoporous Silica Nanoparticles: Synthesis, Biocompatibility and Drug Delivery. Adv. Mater..

[3] Hudson S.P., Padera R.F., Langer R., Kohane D.S. (2008). The biocompatibility of mesoporous silicates. Biomaterials.

[4] Lu J., Liong M., Li Z., Zink J.I., Tamanoi F. (2010). Biocompatibility, Biodistribution, and Drug-Delivery Efficiency of Mesoporous Silica Nanoparticles for Cancer Therapy in Animals. Small.

[5] Wu S.-H., Hung Y., Mou C.-Y. (2011). Mesoporous silica nanoparticles as nanocarriers. Chem. Commun..

[6] He Q., Shi J. (2011). Mesoporous silica nanoparticle based nano drug delivery systems: Synthesis, controlled drug release and delivery, pharmacokinetics and biocompatibility. J. Mater. Chem..

[7] Colilla M., Gonzáleza B., Vallet-Regí M. (2013). Mesoporous silica nanoparticles for the design of smart delivery nanodevices. Biomater. Sci..

[8] Watermann A., Brieger J. (2017). Mesoporous Silica Nanoparticles as Drug Delivery Vehicles in Cancer. Nanomaterials.

[9] Kango S., Kalia S., Celli A., Njuguna J., Habibi Y., Kumar R. (2013). Surface modification of inorganic nanoparticles for development of organic–inorganic nanocomposites—A review. Prog. Polym. Sci..

[10] Li T., Shi S., Goel S., Shen X., Xie X., Chen Z., Zhang H., Li S., Qin X., Yang H. (2019). Recent advancements in mesoporous silica nanoparticles towards therapeutic applications for cancer. Acta Biomater..

[11] Alberti S., Soler-Illia G.J.A.A., Azzaroni O. (2015). Gated supramolecular chemistry in hybrid mesoporous silica nanoarchitectures: Controlled delivery and molecular transport in response to chemical, physical and biological stimuli. Chem. Commun..

[12] Gulzar A., Gaia S., Yanga P., Li C., Ansaric M.B., Linb J. (2015). Stimuli-responsive drug delivery application of polymer and silica in biomedicine. J. Mater. Chem. B.

[13] Niedermayer S., Weiss V., Herrmann A., Schmidt A., Datz S., Müller K., Wagner E., Bein T., Bräuchle C. (2015). Multifunctional polymer-capped mesoporous silica nanoparticles for pH-responsive targeted drug delivery. Nanoscale.

[14] Fullriede H., Abendroth P., Ehlert N., Doll K., Schäske J., Winkel A., Stumpp S.N., Stiesch M., Behrens P. (2016). pH-responsive release of chlorhexidine from modified nanoporous silica nanoparticles for dental applications. BioNanoMaterials.

[15] Bilalis P., Tziveleka L.A., Varlas S., Iatrou H. (2016). pH-Sensitive Nanogates Based on Poly(L-Histidine) for Controlled Drug Release from Mesoporous Silica Nanoparticles. Polym. Chem..

[16] Nairi V., Magnolia S., Piludu M., Nieddu M., Caria C.A., Sogos V., Vallet-Regì M., Monduzzi M., Salis A. (2018). Mesoporous silica nanoparticles functionalized with hyaluronic acid. Effect of the biopolymer chain length on cell internalization. Colloid Surf. B.

[17] Yu E., Galiana I., Martínez-Máñez R., Stroeve P., Marcos M.D., Aznar E., Sancenón F., Murguía J.R., Amorós P. (2015). Poly(N–isopropylacrylamide)–gated Fe_3_O_4_/SiO_2_ core shell nanoparticles with expanded mesoporous structures for the temperature triggered release of lysozyme. Colloid Surf. B.

[18] Chang B., Sha X., Guo J., Jiao Y., Wang C., Yang W. (2011). Thermo and pH dual responsive, polymer shell coated, magnetic mesoporous silica nanoparticles for controlled drug release. J. Mater. Chem..

[19] Yang M.Y., Tan L., Wu H.X., Liu C.J., Zhuo R.X. (2015). Dual-stimuli-responsive polymer-coated mesoporous silica nanoparticles used for controlled drug delivery. J. Appl. Polym. Sci..

[20] Wang F., Xu W., Ouyang Y., Zhang L., Liu H. (2018). Reversible crosslinking terpolymer shell-based mesoporous silica nanoparticles as on-off nanocarriers for pyrene-releasing application. J. Taiwan Inst. Chem. E.

[21] Zhu Y., Shi J., Shen W., Dong X., Feng J., Ruan M., Li Y. (2005). Stimuli-Responsive Controlled Drug Release from a Hollow Mesoporous Silica Sphere/Polyelectrolyte Multilayer Core–Shell Structure. Angew. Chem. Int. Ed..

[22] Zhu Y., Shi J. (2007). A mesoporous core-shell structure for pH-controlled storage and release of water-soluble drug. Micropor. Mesopor. Mater..

[23] Tamanna T., Bulitta J.B., Yu A. (2015). Controlling antibiotic release from mesoporous silica nano drug carriers via self-assembled polyelectrolyte coating. J. Mater. Sci..

[24] Minati L., Antonini V., Dalla Serra M., Speranza G., Enrichi F., Riello P. (2013). pH-activated doxorubicin release from polyelectrolyte complex layer coated mesoporous silica nanoparticles. Microporous Mesoporous Mater..

[25] Feng W., Nie W., He C., Zhou X., Chen L., Qiu K., Wang W., Yin Z. (2014). Effect of pH responsive alginate/chitosan multilayers coating on delivery efficiency, cellular uptake and biodistribution of mesoporous silica nanoparticles based nanocarriers. ACS Appl. Mater. Interfaces.

[26] Du P., Zhao X., Zeng J., Guo J., Liu P. (2015). Layer-by-layer engineering fluorescent polyelectrolyte coated mesoporous silica nanoparticles as pH-sensitive nanocarriers for controlled release. Appl. Surf. Sci..

[27] Wang J., Liu H., Leng F., Zheng L., Yang J., Wang W., Huang C.Z. (2014). Autofluorescent and pH-responsive mesoporous silica for cancer-targeted and controlled drug release. Microporous Mesoporous Mater..

[28] Hu L., Sun H., Zhao Q., Han N., Bai L., Wang Y., Jiang T., Wang S. (2015). Multilayer encapsulated mesoporous silica nanospheres as an oral sustained drug delivery system for the poorly water-soluble drug felodipine. Mater. Sci. Eng. C.

[29] Yilmaz M.D. (2016). Layer-by-layer hyaluronic acid/chitosan polyelectrolyte coated mesoporous silica nanoparticles as pH-responsive nanocontainers for optical bleaching of cellulose fabrics. Carbohydr. Polym..

[30] Anirudhan T.S., Vasantha C.S., Sasidharan A.V. (2017). Layer-by-layer assembly of hyaluronic acid/carboxymethylchitosan polyelectrolytes on the surface of aminated mesoporous silica for the oral delivery of 5-fluorouracil. Eur. Polym. J..

[31] Tsitsilianis C., Papanagopoulos D., Lutz P. (1995). Amphiphilic heteroarm star copolymers of polystyrene and poly(ethylene oxide). Polymer.

[32] Kennemur J.G. (2019). Poly(vinylpyridine) Segments in Block Copolymers: Synthesis, Self-Assembly, and Versatility. Macromolecules.

[33] Gunawidjaja R., Peleshanko S., Genson K.L., Tsitsilianis C., Tsukruk V.V. (2006). Monomolecular micelles Surface morphologies of Langmuir-Blodgett monolayers of PEO_n_PS_n_ multiarm star copolymers. Langmuir.

[34] Xu W., Ledin P.A., Iatridi Z., Tsitsilianis C., Tsukruk V.V. (2016). Multicompartmental Microcapsules with Orthogonal Programmable Two-Way Sequencing of Hydrophobic and Hydrophilic Cargo Release. Angew. Chem. Int. Ed..

[35] Akagawa M., Suyama K. (2001). Amine oxidase-like activity of polyphenols. Mechanism and properties. Eur. J. Biochem..

[36] Shutava T.G., Balkundi S.S., Vangala P., Steffan J.J., Bigelow R.L., Cardelli J.A., O’Neal D.P., Lvov Y.M. (2009). Layer-by-Layer-Coated Gelatin Nanoparticles as a Vehicle for Delivery of Natural Polyphenols. ACS Nano.

[37] Daglia M. (2012). Polyphenols as antimicrobial agents. Curr. Opin. Biotechnol..

[38] Chung K.T., Wong T.Y., Wei C.I., Huang Y.W., Lin Y. (1998). Tannins and Human Health: A Review. Crit. Rev. Food Sci. Nutr..

[39] Shutava T., Prouty M., Kommireddy D., Lvov Y. (2005). pH Responsive Decomposable Layer-by-Layer Nanofilms and Capsules on the Basis of Tannic Acid. Macromolecules.

[40] Kozlovskaya V., Kharlampieva E., Drachuk I., Cheng D., Tsukruk V.V. (2010). Responsive microcapsule reactors based on hydrogen-bonded tannic acid layer-by-layer assemblies. Soft Matter.

[41] Zhou L., Chen M., Tian L., Guan Y., Zhang Y. (2013). Release of Polyphenolic Drugs from Dynamically Bonded Layer-by-Layer Films. ACS Appl. Mater. Interfaces.

[42] Liu F., Kozlovskaya V., Zavgorodnya O., Martinez-Lopez C., Catledge S., Kharlampieva E. (2014). Encapsulation of anticancer drug by hydrogen bonded multilayers of tannic acid. Soft Matter.

[43] Chen J., Kozlovskaya V., Goins A., Campos-Gomez J., Saeed M., Kharlampieva E. (2013). Biocompatible Shaped Particles from Dried Multilayer Polymer Capsules. Biomacromolecules.

[44] Kim B.S., Lee H.I., Min Y., Poon Z., Hammond P.T. (2009). Hydrogen-bonded multilayer of pH-responsive polymeric micelles with tannic acid for surface drug delivery. Chem. Commun..

[45] Liang H., Pei Y., Li J., Xiong W., He Y., Liu S., Li Y., Li B. (2016). pH-Degradable antioxidant nanoparticles based on hydrogen-bonded tannic acid assembly. RSC Adv..

[46] Tang C., Sosa C., Pagels R.F., Priestley R.D., Prud’homme R.K. (2016). Efficient preparation of size tunable PEGylated gold nanoparticles. J. Mater. Chem. B.

[47] Kosmilski M. (2004). pH-dependent surface charging and points of zero charge II. Update. J. Colloid Interface Sci..

[48] Choi I., Malak S.T., Xu W., Heller W.T., Tsitsilianis C., Tsukruk V.V. (2013). Multicompartmental microcapsules from star copolymer micelles. Macromolecules.

