# Supplementary material for: Multicompartmental Mesoporous Silica/Polymer Nanostructured Hybrids: Design Capabilities by Integrating Linear and Star-Shaped Block Copolymers
Source: Polymers (Basel). 2019 Dec 31;12(1):51. doi: 10.3390/polym12010051 (PMC7023666; doi:10.3390/polym12010051)
Supplement: Supplementary file 1 [file polymers-12-00051-s001.pdf]

polymers

MDPI

Supporting Information

Multicompartmental Mesoporous Silica/Polymer Nanostructured Hybrids: Design Capabilities by Integrating Linear and Star-shaped Block Copolymers

Synthesis and Characterization of copolymers

The poly(2-vinylpyridine)-poly(ethylene oxide) (P2VP-b-PEO) block copolymer and the $PS_{10}PEO_{10}$ heteroarm star copolymer were synthesized by anionic "living" polymerization and sequential addition of monomers. Their molecular weight characteristics are presented in Table S1

Table S1. Molecular characteristics of the copolymers.

polymer	Composition (PEO wt %)[a]	Molecular weight: Mw (g/mol)	Polydispersity index: Đ [b]
		**P2VP-b-PEO**	
P2VP		3820 [b]	1.25
PEO		17050[c]	
P2VP-b-PEO	81,7	20870[d]	1.06
		**$PS_{10}PEO_{10}$**	
PSarm		3100[b]	
PS_{10} (star)		34200[e]	
$PS_{10}PEO_{10}$ (star)	88.0	285000[f]	
PEOarm		25080[c]	

[a] by ^{1}H-NMR, [b] by SEC (size exclusion chromatography).[c]calculated, [d] calculated by equation 1, [e] calculated by static light scattering, [f] calculated by equation 2.

$$M_{w,P2VP\text{-}PEO} = M_{w,P2VP}\ (1/1\text{-}w_{PEO}) \qquad eq.\ 1$$

$$M_{w,PS_{10}PEO_{10}} = M_{w,PS_{10}}\ (1/1\text{-}w_{PEO}) \qquad eq.\ 2$$

Details of the synthesis and characterization of the copolymers are reported elsewhere (i.e. P2VP-b-PEO [1], $PS_{10}PEO_{10}$ [2]).

Table S2. Characteristics of the SiO_2 particles.

Size (μm)	2.25±0.25
Pore volume ($cm^3 * g^{-1}$)	0.2-0.4
Pore size (nm)	4
Surface ($m^2 * g^{-1}$)	300-400

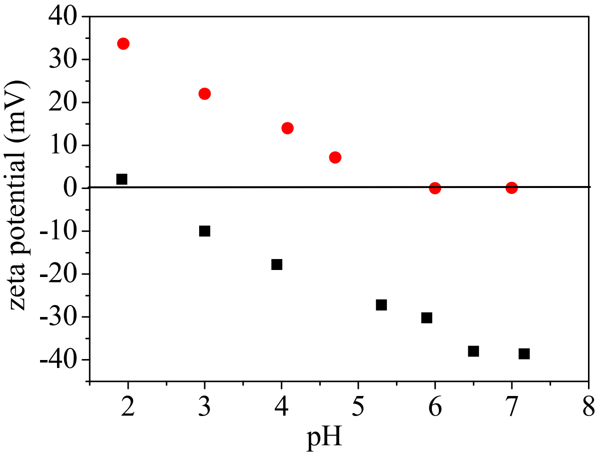

Figure S1. Zeta potential of aqueous dispersion of SiO_2 (■) and aqueous P2VP-b-PEO solutions (●) at different pH.

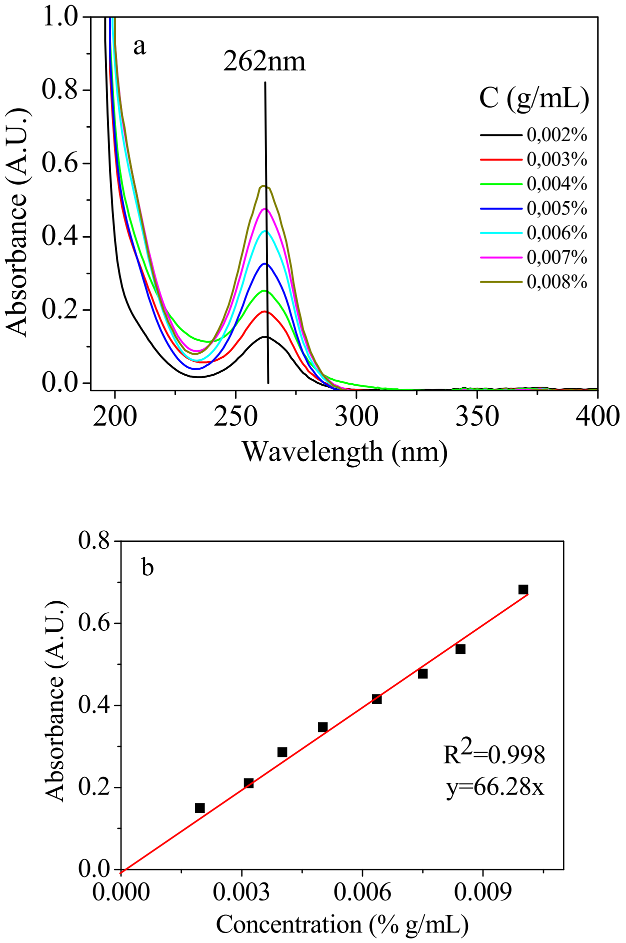

Figure S2. a) UV-Vis spectra of aqueous P2VP-b-PEO solutions at a fixed pH 3.0; b) calibration curve of the P2VP-b-PEO at pH 3.0. .

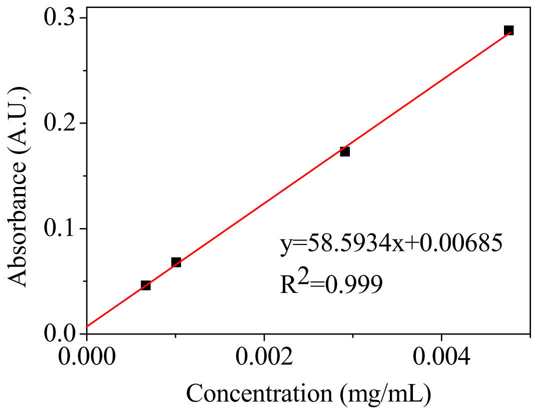

Figure S3. Calibration curve of calcein in water.

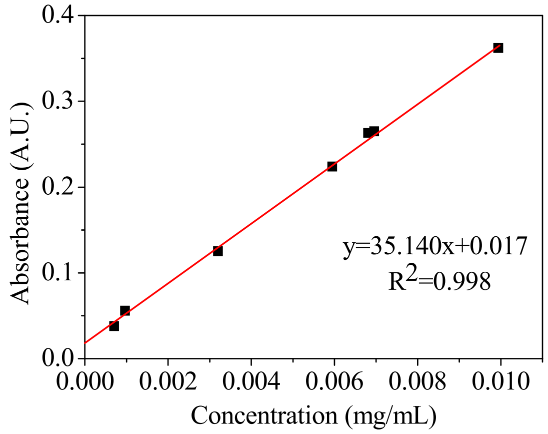

Figure S4. Calibration curve of calcein in PB buffer pH 5.0.

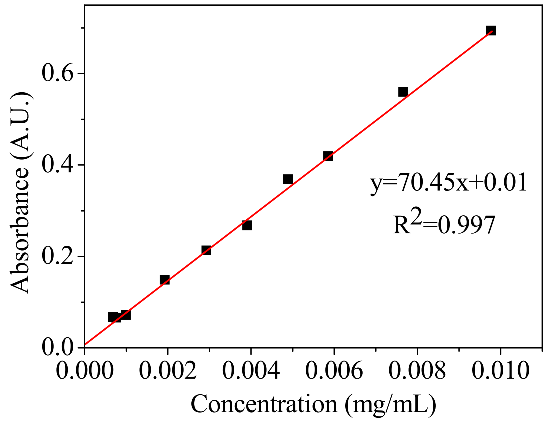

Figure S5. Calibration curve of calcein in PB buffer pH 6.0.

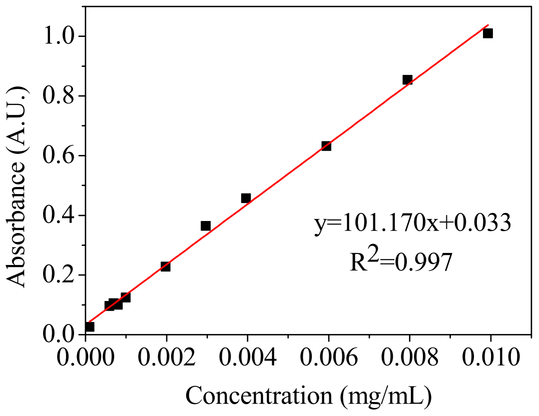

Figure S6. Calibration curve of calcein in PB buffer pH 7.4.

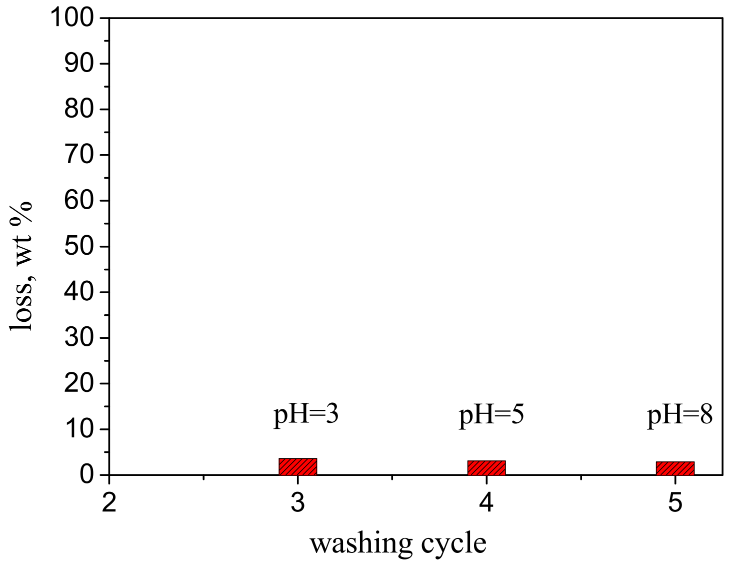

Figure S7. Loss (wt %) of adsorbed copolymer versus washing cycle (redispertion-stirring-centrifugation-separation) at various pH.

References

1. Tsitsilianis, C.; Alexandridis, P.; Lindman, B. Lyotropic liquid crystalline structures formed by amphiphilic heteroarm star copolymers. *Macromolecules* **2001**, *34*, 5979–5983.
2. Liaskoni, A.; Angelopoulou, A.; Voulgari, E.; Popescu, M.-T.; Tsitsilianis, C.; Avgoustakis. K. Paclitaxel controlled delivery using a pH-responsive functional-AuNP/block-copolymer vesicular nanocarrier composite system. *Eur. J. Pharmac. Sci.* **2018**, *113*, 177–186.

© 2020 by the authors. Submitted for possible open access publication under the terms and conditions of the Creative Commons Attribution (CC BY) license (http://creativecommons.org/licenses/by/4.0/).
